# Exposure to high endotoxin concentration increases wheezing prevalence among laboratory animal workers: a cross-sectional study

**DOI:** 10.1186/s12890-016-0233-1

**Published:** 2016-05-06

**Authors:** Amanda Souza Freitas, Christian Silva Simoneti, Erica Ferraz, Ericson Bagatin, Izaira Tincani Brandão, Celio Lopes Silva, Marcos Carvalho Borges, Elcio Oliveira Vianna

**Affiliations:** Department of Social Medicine, Medical School of Ribeirão Preto, University of São Paulo, Ribeirão Preto, Brazil; Department of Medicine, Medical School of Ribeirão Preto, University of São Paulo, Ribeirão Preto, Brazil; Department of Preventive and Social Medicine, State University of Campinas, Campinas, Brazil; Department of Biochemistry and Immunology, Medical School of Ribeirão Preto, University of São Paulo, Ribeirão Preto, Brazil

**Keywords:** Endotoxin, Wheezing, Occupational disease, Animal handler

## Abstract

**Background:**

Endotoxin from Gram-negative bacteria are found in different concentrations in dust and on the ground of laboratories dealing with small animals and animal houses.

**Methods:**

Cross-sectional study performed in workplaces of two universities. Dust samples were collected from laboratories and animal facilities housing rats, mice, guinea pigs, rabbits or hamsters and analyzed by the “*Limulus amebocyte lysate*” (LAL) method. We also sampled workplaces without animals. The concentrations of endotoxin detected in the workplaces were tested for association with wheezing in the last 12 months, asthma defined by self-reported diagnosis and asthma confirmed by bronchial hyperresponsiveness (BHR) to mannitol.

**Results:**

Dust samples were obtained at 145 workplaces, 92 with exposure to animals and 53 with no exposure. Exposed group comprised 412 subjects and non-exposed group comprised 339 subjects. Animal-exposed workplaces had higher concentrations of endotoxin, median of 34.2 endotoxin units (EU) per mg of dust (interquartile range, 12.6–65.4), as compared to the non-exposed group, median of 10.2 EU/mg of dust (interquartile range, 2.6–22.2) (*p* < 0.001). The high concentration of endotoxin (above whole sample median, 20.4 EU/mg) was associated with increased wheezing prevalence (*p* < 0.001), i.e., 61 % of workers exposed to high endotoxin concentration reported wheezing in the last 12 months compared to 29 % of workers exposed to low endotoxin concentration. The concentration of endotoxin was not associated with asthma report or with BHR confirmed asthma.

**Conclusion:**

Exposure to endotoxin is associated with a higher prevalence of wheezing, but not with asthma as defined by the mannitol bronchial challenge test or by self-reported asthma. Preventive measures are necessary for these workers.

## Background

Endotoxin is a constituent of the outer cell wall of Gram-negative bacteria and its main component is lipopolysaccharide (LPS) [1]. Thorne et al. [2] showed that exposure to endotoxin in the domestic environment significantly increases the prevalence of asthma in adults. Endotoxin has also been related to the onset of “Monday morning asthma” among cotton workers, to damp fever, grain fever, toxic pneumonia, and acute systemic effects such as malaise and fever [3]. Adults exposed to high endotoxin levels in house dust (more than 11.2 EU/mg of dust) are more susceptible to worsening of allergic and non-allergic asthma and to the development of asthma [4–6].

Although few studies are available, some authors have shown that exposure to high endotoxin concentrations is associated with an increased risk of wheezing during the first year of life [7, 8]. On the other hand, some studies have suggested that early exposure to endotoxin present in the environment has a protective effect on the risk of atopic sensitization in children and possibly also on the population of workers exposed to high endotoxin levels [3, 8, 9].

Researchers and technicians who work with laboratory animals are exposed to both animal allergens and endotoxin in the workplace. Some studies have identified the agents present in organic dirt in the workplace and their interaction with respiratory and allergic diseases among the workers. These studies have evaluated various environments and populations [9–12], but few of them have dealt with workers exposed to laboratory animals regarding the interaction of endotoxin with respiratory symptoms [13, 14].

In the workplace, the concentration of endotoxin responsible for triggering respiratory effects (including asthma) is often below the permissible exposure limits (PELs) or occupational exposure limits (OELs) [15], but more studies are needed on the role of endotoxin in the workplace and on the genesis of risks for workers. Although several negative effects have been reported, there are also beneficial effects of exposure to endotoxin [7, 9] and more studies are needed to clarify such effects. On this basis, we formulated the hypothesis that exposure to endotoxin may cause asthma symptoms. Thus, the objective of the present study was to determine the association between the quantity of endotoxin detected in the workplace and the presence of asthma or wheezing.

## Methods

This is a cross-sectional study that was conducted at two universities, the University of São Paulo (USP) at Ribeirão Preto and State University of Campinas (UNICAMP), São Paulo State, Brazil. Sample selection and study protocol have been previously described [16]. Volunteers were workers or students with exposure to laboratory animals (exposed group) and without exposure (non-exposed group).

Laboratories and workplaces were randomly selected from the facilities and 145 workplaces were included in this study, 53 workplaces belonged to the non-exposed group (339 subjects) and 92 workplaces to the exposed group (412 subjects). At least 90 % of subjects in every workplace consented to participate; the overall consent and participation rate was 95 %. The study was reviewed and approved by the Ethics Committees of both institutions: Medical School of Ribeirão Preto, University of S. Paulo (*Comitê de Ética em Pesquisa do FMRP-USP*, protocol number 9428/2009), and the School of Medical Sciences, State University of Campinas (*Comitê de Ética em Pesquisa da UNICAMP*, protocol number 779/2009). Written informed consent was obtained from all subjects after reading and discussing the protocol individually.

### Questionnaires and diagnostic procedures

Questionnaires were applied to 751 subjects with questions inquiring about personal history of allergic diseases, pet owning history, smoking, respiratory, nasal, ocular, and skin symptoms. The questionnaire items also included job characteristics such as duration of working with laboratory animals, job titles, job contents, frequency of contact with animals, species, time spent handling animals, use of protective equipment, asthma and rhinitis symptoms. Subjects also underwent skin prick test, spirometry and bronchial hyperresponsiveness (BHR) test with mannitol. For most questions about symptoms and risk factors, we used questions from the European Community Respiratory Health Survey questionnaire, translated into Portuguese, adapted to the Brazilian lexicon and previously tested [17, 18].

### Skin prick test

Skin prick tests (SPTs) were carried out following the recommendations of the European Academy of Allergology and Clinical Immunology [19]. Subjects did not take antihistamine drugs for 15 days prior to SPT. The test was considered positive when a reaction led to a wheal diameter of at least 3 mm in the absence of a reaction to physiological saline solution and in the presence of a positive reaction to histamine. The allergens included common allergens (*Dermatophagoides farinae, Dermatophagoides pteronyssinus*, *Felis domesticus*, *Canis familiaris*, *Blomia tropicalis, Blattella germanica*, *Periplaneta americana*, *Alternaria alternata*, *Aspergillus fumigatus, Cladosporium herbarum*, and mixed grass) and extracts from animals (mouse, rat, hamster, guinea pig and rabbit). These were called occupational allergens.

### Spirometry

The lung function was analyzed using a Koko spirometer and software (PDS Instrumentation, Inc., Louisville, Colorado, USA), the spirometer were calibrated every day. Lung function was conducted in the sitting position with the volunteers using a nose clip. We performed at most 8 attempts to obtain at least 3 technically satisfactory maneuvers for each volunteer. If after 8 attempts, at least 3 technically satisfactory maneuvers were not obtained, lung function test was interrupted [20]. The reference values of Crapo et al. [21] were used.

### Bronchial challenge test with mannitol

Dry powdered mannitol (Aridol) was supplied in kit form (Pharmaxis Ltd., New South Wales, Australia), which contained 1 empty capsule (0 mg), capsules containing 5, 10 and 20 mg, and 15 capsules containing 40 mg (cumulative dose of 635 mg). The challenge began with the empty capsule, followed by inhalation of 5, 10, 20, 40, 80, 160, 160, and 160 mg dry powdered mannitol. Forced expiratory volume in one second (FEV1) was measured 60 s after every inhalation. This procedure was repeated for each dose step until a 15 % fall in FEV1 was achieved or the cumulative dose reached 636 mg. A positive test (BHR) was defined as a 15 % decrease in FEV1 to 635 mg or less [22].

### Variables

Groups: The exposed group was composed of volunteers exposed to laboratory animal, including: researchers, technicians and students. The non-exposed group consisted of secretaries, car drivers, management employees, computer technicians, students and other employees who had no contact with laboratory animals. For more details on characteristics of groups and subjects, refer to Ferraz et al. [16].

Self-reported asthma was defined by positive answer to the question: Have you ever had asthma?

Confirmed asthma: A volunteer was considered an asthmatic if he/she had a positive bronchial challenge test and had experienced symptoms of wheezing, dyspnea during the night or at day in the previous 12 months, or tightness of the chest during the night.

Current wheezing was defined by a positive answer to the question: “Have you had wheezing or whistling in your chest any time in the last 12 months?”

Past exposure to laboratory animals was defined by a positive answer to the question: “In the past, did you work in places with animals (laboratory or animal rooms)?”

Pet ownership: This variable was defined by the question “In the last 12 months, have you had pets at home?”

Endotoxin concentration: High endotoxin concentrations included values above the median (20.4 EU/mg) and low endotoxin concentrations means ≤ 20.4 EU/mg. This cut-off is the median of endotoxin concentration of all the workplaces, i.e., both exposed and non-exposed group workplaces (whole sample median).

Smoking: This variable was defined by a positive answer to the question “Have you smoked for more than one year?”

Atopy: This variable was defined by a positive skin prick test to any allergen.

### Dust samples

Floor dust samples were collected at both groups workplaces. One square meter was sampled for 2 min using a vacuum cleaner (Arno Nitro, São Paulo, Brazil) equipped with a fiberglass filter with a pore size of approximately 1 μm. Filters were pre- and post-weighed. Loaded filters were transported separately, packed and stored in a refrigerator (2 to 8 °C) until processing within a maximum of 4 weeks. Amount of dust varied in different workplaces. This amount was compared between groups and concentration of endotoxin was expressed in relation to dust weight.

Filters with dust were transferred to 50 ml pyrogen-free tubes. Dust was extracted in 35 ml of pyrogen-free water containing 0.05 % (v/v) Tween-20. After an ultrasound bath for 20 min at 40 kHz frequency (Ultra Sonic Cleaner, Unique, São Paulo, Brazil), the tubes were centrifuged for 10 min at 991xg, 1 ml of the extract was pipetted and again centrifuged at 1000xg for 10 min at room temperature, and the supernatant was stored in 5 μl aliquots.

### Endotoxin analysis

Endotoxin concentration was measured using the kinetic chromogenic *Limulusamebocyte lysate* assay (LAL - Bio Whittaker - A CAMBREX Company). Briefly, 100 μl of the sample was incubated for 10 min at 37 °C in a 96-well microtiter plate. The LAL reagent was reconstituted in pyrogen-free water, rapidly added to the samples, and kinetics was recorded with a temperature-controlled microplate reader at 405 nm and 37 °C. Endotoxin levels are reported as concentration [endotoxin unit/mg of collected dust (EU/mg)].

### Statistical analysis

Univariate analysis with chi-square test was used to compare prevalence estimates and proportions between groups (exposed versus non-exposed group), between different asthma definitions and to compare wheezing prevalence among endotoxin concentration quartiles. Two-tailed Student’s *t* test was used to compare continuous variables (age, dust weight, and endotoxin concentration) between exposed and non-exposed groups.

Multivariate analysis was performed to test for associations between endotoxin levels and asthma or wheezing by using a modified Poisson regression approach, i.e., Poisson regression with a robust error variance [23]. Endotoxin levels were categorized according to the median in high and low concentration levels. The model was adjusted using the PROC GENMOD procedure of the SAS software version 9.2, which was also used for data analysis.

## Results

A total of 751 volunteers participated in this study, with 412 volunteers in the exposed group and 339 volunteers in the non-exposed group. The mean age of the volunteers was 29 years, without difference between groups. Female predominance was observed (*p* < 0.001). We collected 145 samples of dust, 92 samples in the exposed group and 53 in the non-exposed group. The concentration of endotoxin in the dust was significantly different between groups. In the exposed group, the median concentration of endotoxin was 34.2 EU/mg of dust; and, in the non-exposed group, it was 10.2 EU/mg of dust (*p* < 0.001). In the exposed group, there was a higher prevalence of students (*p* < 0.001) (Table 1).

**Table 1:** Subjects and workplaces characteristics

Variables	Total *n* = 751	Exposed group *n* = 412	Non-exposed group *n* = 339	*p* value
Workplaces	145	92	53	
Age (years)	29 (25–40)	29 (25–37)	30 (25–41)	0.14
Female, n (%)	446 (59.3 %)	221 (53.6 %)	225 (66.3 %)	<0.001*
Dust (g/m^2^)	0.112 (0.04–0.31)	0.110 (0.02–0.23)	0.117 (0.05–0.31)	0.63
Endotoxin (EU/mg of dust)	20.4 (6.6–45.5)	34.2 (12.6–65.4)	10.2 (2.6–22.2)	<0.001*
Technicians /administrators	326 (43.4 %)	140 (34.0 %)	186 (54.9 %)	<0.001*
Students	360 (48.0 %)	240 (58.2 %)	120 (35.4 %)	
Researchers	65 (8.6 %)	32 (7.8 %)	33 (9.7 %)	

Results are expressed as median (interquartile range) or n (%)

*chi-square test

*EU* endotoxin unit

There was no difference in the prevalence of sensitization to common allergens between the groups. The prevalence of sensitization to occupational allergens was significantly higher in the exposed group (16.5 %) compared to the non-exposed group (2.6 %) (*p* < 0.001).). The prevalence of self-reported asthma, confirmed asthma and current wheezing were not different between the groups. A significant difference was found for past exposure to laboratory animals: 70 % for the animal exposed group and 30 % for the non-exposed group (*p* < 0.001) (Table 2).

**Table 2:** Clinical data and diagnoses

Variables	Total *n* = 751	Exposed group *n* = 412	Non-exposed group *n* = 339	*p* value
Skin prick test				
Commom allergen (s)	333 (44.3 %)	176 (42.7 %)	157 (46.3 %)	0.269
Occupational allergen (s)	77 (10.2 %)	68 (16.5 %)	9 (2.6 %)	<0.001*
Smoking	129 (17.1 %)	72 (17.4 %)	57 (16.8 %)	0.811
Spirometry (normal)	715 (94.7 %)	389 (94.4 %)	326 (96.1 %)	0.265
Self-reported asthma	88 (12.0 %)	48 (11.6 %)	40 (11.8 %)	1.00
Confirmed asthma	73 (10 %)	42 (10 %)	31 (9 %)	0.71
Wheezing	163 (21.7 %)	96 (23.3 %)	67 (19.7 %)	0.24
BHR^a^	95 (12.9 %)	57 (14.0 %)	38 (11.5 %)	0.31
Past exposure to laboratory animals	240 (32 %)	168 (70 %)	72 (30 %)	<0.001*
Pet owning history	485 (64.6 %)	258 (53.2 %)	227 (46.8 %)	0.22

Results are expressed as median (interquartile range) or n (%). *chi-square test. *BHR* bronchial hyperresponsiveness

^a^16 subjects did not undergo bronchial challenge test, 6 in the exposed group and 10 in the non-exposed group

**Fig. 1:**
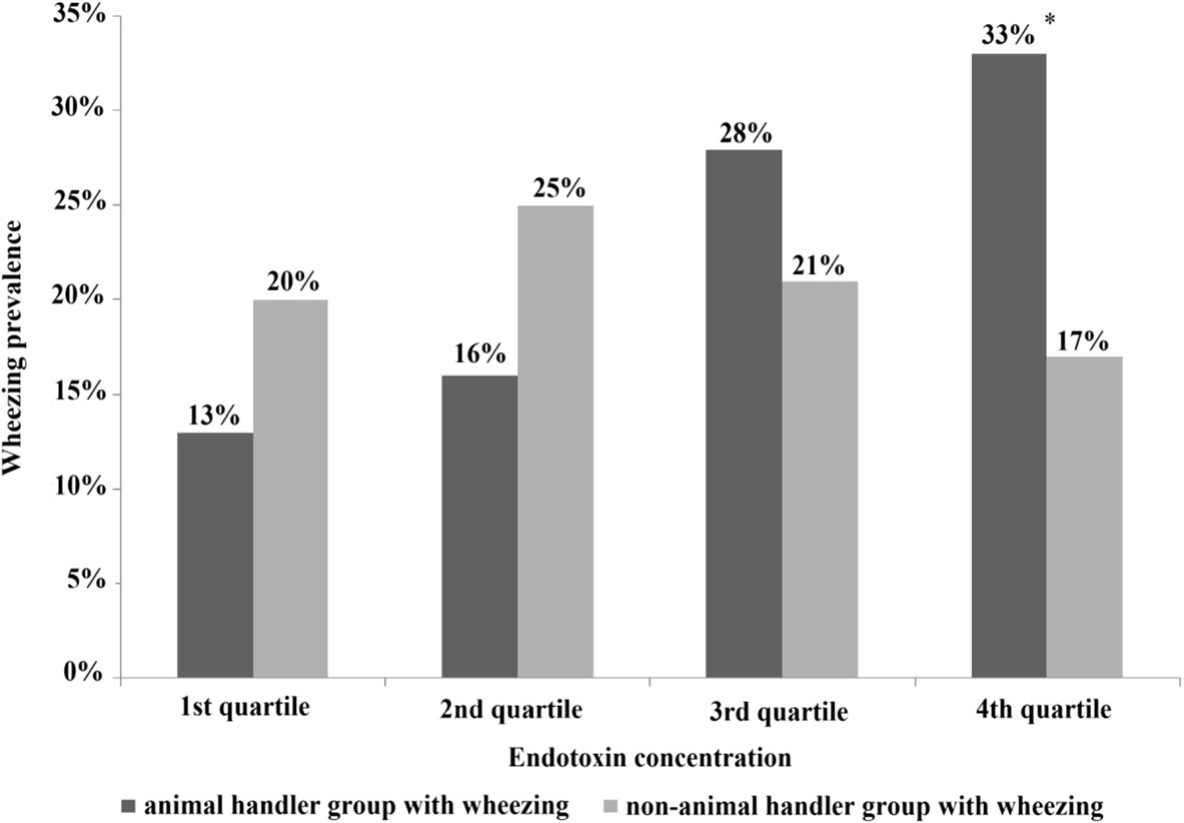
Wheezing prevalence according to endotoxin concentration in both groups. *p < 0.01 for the comparison of wheezing prevalence among levels of endotoxin concentration in the animal exposed group. Wheezing was a positive answer to the question: Have you had wheezing or whistling in your chest any time in the last 12 months?

In the multivariate analyses, no associations were found between high levels (above whole sample median) of endotoxin (>20.4 EU/mg) and self-reported asthma (Table 3) or asthma confirmed by BHR (Table 4). We found association between wheezing and high levels of endotoxin (>20.4 EU/mg) (Table 5). When endotoxin concentrations were divided into 4 categories (quartiles), wheezing prevalence increased with increasing endotoxin concentration in the exposed group (Fig. 1).

**Table 3:** Evaluation of risk factors for self-reported asthma

Variables	Categories	Reported asthma							
		Crude model				Adjusted model			
		PR	95 % CI		*p* value	PR	95 % CI		*p* value
Group	Exposed vs Non-exposed	0.98	0.66	1.47	0.94	0.99	0.67	1.47	0.96
Endotoxin levels	Low vs High	0.99	0.66	1.47	0.95	1.01	0.68	1.50	0.96
Institutions	UNICAMP vs USP	1.01	0.68	1.50	0.97	1.06	0.73	1.56	0.76
Atopy	Yes vs No	7.93	4.29	14.68	<0.01	7.69	4.18	14.16	<0.01
Smoking	Yes vs No	0.88	0.51	1.54	0.67	0.84	0.49	1.43	0.51
Sex	Male vs Female	1.09	0.72	1.63	0.69	1.15	0.77	1.73	0.49
Past exposure to laboratory animals	Yes vs No	1.41	0.94	2.11	0.10	1.26	0.86	1.84	0.24
Pet owning history	Yes vs No	1.19	0.77	1.82	0.43	1.13	0.76	1.70	0.54
Age	Continuous	0.99	0.97	1.01	0.16	1.01	0.99	1.04	0.21
Worker functions	Technicians/secretaries vs researchers	7.20	1.01	51,54	0.05	6.75	0.92	49.36	0.06
	Students vs researchers	8.83	1.24	62.78	0.03	7.73	1.02	58.47	0.05

*PR* prevalence ratio, *CI* confidence interval, *UNICAMP* State University of Campinas, *USP* University of São Paulo; Low endotoxin concentration: ≤ 20.4 EU/mg of dust; High endotoxin concentration: > 20.4 EU/mg of dust

**Table 4:** Evaluation of risk factors for asthma confirmed

Variables	Categories	Asthma confirmed by BHR							
		Crude model				Adjusted model			
		PR	95 % CI		*p* value	PR	95 % CI		*p* value
Group	Exposed vs Non-exposed	1.08	0.69	1.68	0.73	1.12	0.71	1.76	0.63
Endotoxin levels	Low vs High	1.21	0.78	1.88	0.40	0.79	0.51	1.22	0.29
Institutions	UNICAMP vs USP	1.40	0.89	2.18	0.14	1.25	0.81	1.93	0.31
Atopy	Yes vs No	7.07	3.68	13.58	<0.01	6.60	3,42	12.71	<0.01
Smoking	Yes vs No	0.89	0.48	1.64	0.70	1.00	0.54	1.86	0.99
Sex	Male vs Female	1.39	0.87	2.21	0.17	1.48	0.93	2.37	0.10
Past exposure to laboratory animals	Yes vs No	1.36	0.87	2.14	0.17	1.24	0.79	1.94	0.35
Pet owning history	Yes vs No	1.17	0.73	1.87	0.52	1.05	0.66	1.66	0.83
Age	Continuous	0.96	0.94	0.99	<0.01	0.98	0.95	1.01	0.16
Worker functions	Technicians/secretaries vs researchers	1.44	0.52	3.95	0.48	1.35	0.49	3.72	0.56
	Students vs researchers	1.74	0.65	4.71	0.27	1.09	0.41	3.72	0.56

*BHR* bronchial hyperresponsiveness, *PR* prevalence ratio, *CI* confidence interval, *UNICAMP* State University of Campinas, *USP* University of São Paulo, Low endotoxin concentration: ≤ 20.4 EU/mg of dust, High endotoxin concentration: > 20.4 EU/mg of dust

**Table 5:** Evaluation of risk factors for wheezing in the last 12 months

Variables	Categories	Wheezing							
		Crude model				Adjusted model			
		PR	95 % CI		*p* value	PR	95 % CI		*p* value
Group	Exposed vs Non-exposed	1.18	0.89	1.55	0.24	1.03	0.78	1.37	0.81
Endotoxin levels	Low vs High	1.44	1.09	1.90	<0.01	1.49	1.14	1.96	<0.01
Institutions	UNICAMP vs USP	0.97	0.74	1.28	0.85	0.89	0.68	1.16	0.38
Atopy	Yes vs No	3.38	2.46	4.65	<0.01	3.20	2.33	4.39	<0.01
Smoking	Yes vs No	0.87	0.59	1.29	0.49	0.94	0.65	1.37	0.74
Sex	Male vs Female	1.06	0.80	1.39	0.70	1.10	0.84	1.44	0.49
Past exposure to laboratory animals	Yes vs No	1.32	1.00	1.73	0.05	1.21	0.93	1.57	1.15
Pet owning history	Yes vs No	1.39	1.02	1.88	0.04	1.38	1.03	1.85	0.03
Age	Continuous	0.97	0.96	0.99	<0.01	0.98	0.96	0.99	0.03
Worker functions	Technicians/secretaries vs researchers	1.34	0.73	2.46	0.35	1.08	0.60	1.94	0.80
	Students vs researchers	1.66	0.91	3.01	0.10	0.93	0.52	1.69	0.82

*PR* prevalence ratio, *CI* confidence interval, *UNICAMP* State University of Campinas, *USP* University of São Paulo, Low endotoxin concentration: ≤ 20.4 EU/mg of dust, High endotoxin concentration: > 20.4 EU/mg of dust

Among the workers who reported asthma in response to the questionnaire, 62 (72 %) also reported wheezing in the last 12 months (p ≤ 0.01) and among the workers with confirmed asthma, 64 (91 %) also reported wheezing (p ≤ 0.01). Among individuals who reported wheezing and asthma (*n* = 62), there was no association between wheezing and high endotoxin concentration in dust (*p* = 0.914). In cases of wheezing excluding asthma (*n* = 80), there was association of wheezing with high endotoxin concentrations (*p* = 0.001).

## Discussion

We confirmed the presence of endotoxin in dust collected from workplaces that had laboratory animals. Detectable levels of endotoxin were found also in workplaces without laboratory animals. However, the amount of endotoxin was higher in the exposed group (34.2 EU/mg) vs non-exposed group (10.2 EU/mg) (*p* < 0.001).

In the exposed group, workplaces with high concentrations of endotoxin (above whole sample median) were more frequent in those dealing with rats (79 % of the workplaces) than in those dealing with mice (27 %, *p* < 0.001 - data not shown in Results). Low doses of endotoxin were more frequent in the workplaces of the non-exposed group (66 % versus 36 % of the exposed group). This showed that the amount of endotoxin was associated with the presence of animals in the workplaces and probably other animal products that may pose risks to workers.

Analysis of the relationship between endotoxin concentration and report of respiratory symptoms or diseases among workers showed that high concentrations of endotoxin collected from the floor of laboratories and animal rooms were associated with wheezing in the last 12 months. However, we did not find an association with either reported asthma or confirmed asthma.

Thus, the concentration of endotoxin was associated with wheezing, but not with asthma, leading us to question if wheezing is due to asthma or to other pathophysiological mechanisms [24]. The association between wheezing and asthma, very well known in the medical literature, was recognized in the present study. Wheezing could be an initial manifestation of an endotoxin-dependent asthma, but not yet diagnosed as asthma (therefore, not reported by the subjects). Wheezing could also be related to intermittent asthma - without constant hyperresponsiveness. In this case, hyperresponsiveness would not be detected on the day of examination.

Our results showed an increase in reported wheezing with increasing endotoxin concentrations in the animal exposed group, whereas this relationship was not detected in the non-exposed group. Oldenburg et al. [25] showed an exposure-response relationship between occupational inhalative endotoxin exposure and obstructive ventilation patterns in German cotton textile workers. A significant decrease for FEV1/FVC rate was detected in association with increasing exposure to airborne endotoxin measured in 22 different workplaces of the cotton-spinning mill.

However, it is difficult to determine whether endotoxin in environments with animals directly causes wheezing, or if there is some interaction with other allergenic proteins present in the environment that may cause increased wheezing. If allergens or other animal products were directly responsible for the increase of wheezing, endotoxin could be only a marker of the presence of animals. In this work, allergen concentration in dust was measured and no association with symptoms, asthma or BHR has been detected [26]. It seems, thus, that the observed association between endotoxin levels and wheeze is not due to laboratory animal allergy rather than to endotoxin.

Our results are similar to those previously reported regarding increased endotoxin exposure related to animal laboratories [14]. Higher quantities of endotoxin were detected in the air of animal research laboratories than in laboratories using no animals and in areas outside the animal facilities. This suggests that mice and cages kept in the laboratories may have been the source of endotoxin. Concentrations of air-transported endotoxin were detected in 100 % of the samples collected, where as mouse allergens were detected in only 70 % of the samples, probably because there were other sources of endotoxin in addition to mice.

The association between exposure to endotoxin and wheezing among farmers differs according to CD14 type (a cell differentiation marker that serves as a link between LPS and macrophages) [27]. G and T alleles (21.619 A/G and 2159 C/T polymorphisms, respectively) are associated with higher circulating CD14 levels, whereas the T allele (CD14/2550 T) is associated with lower plasma-soluble CD14 levels [28, 29]. Individuals who carry the CD14/-260 C allele are more responsive to endotoxin than those who carry the homozygous T allele even in the presence of high occupational levels. The dose-response curve for individuals with CD14/-260 CT and CC demonstrated wheezing in the presence of high occupational levels of endotoxin. Pacheco et al. [30] published a study with similar findings, although regarding a population of workers exposed to laboratory animals. Workers carrying the CD14/1619 G allele and exposed to high endotoxin concentrations (fourth quartile) had a significantly lower pulmonary function than workers similarly exposed but carrying the AA genotype.

Skogstad et al. [31] analyzed the effects of exposure to bacterial endotoxin in 28 workers employed by a factory that produced bioproteins derived from a bacterial species (*Methylococcus capsulatum*). The study lasted 5 years, 4 of them during exposure and 1 after the cessation of exposure. Forced vital capacity values obtained by spirometry were significantly lower in the group exposed to low endotoxin levels compared to the values obtained in the year without exposure. The values of FEV1 were significantly higher after the cessation of exposure than during exposure in the group with low exposure. There was also a significant association between exposure to high endotoxin concentrations and number of leukocytes detected in blood tests, with leukocyte values normalizing after the cessation of exposure. These findings reveal the association between exposure and inflammatory activity in the workers’ lungs. Endotoxin inhalation can induce a systemic inflammatory response by macrophage activation and by neutrophil influx into the lungs. An increase in IL-6 was observed during exposure and the endotoxin-induced increase in blood leukocytes and D-dimer was reversed one year after the factory was closed. Since endotoxin was found to be present in the workers’ blood, a direct stimulation of circulating cells of the immunological system seems to be possible. Consequently, both mechanisms may result in an increase of acute phase proteins.

A peculiarity of the present study was collection of dust from the floor. The sampling of endotoxin in the dust deposited on the floor rather than the sampling of airborne endotoxin has the advantage of easy and standardized collection. Indeed, the sampling of airborne endotoxin is a complex procedure for which, to date, there is no fully standardized protocol [32].

Limitations of this study may be a cross-sectional design instead of longitudinal one, lack of evaluation of airborme endotoxin and lack of evaluation of other environmental variables, such as room ventilation and cleaning schedule.

We believe that the study of components present in the workplace, such as endotoxin, and their role in the triggering of allergic and respiratory diseases among the workers could be the first step of a health care program for these workers. Since exposure to high quantities of endotoxin is associated with a more frequent report of wheezing among workers exposed to laboratory animals, it is necessary to propose intervention measures in order to reduce this exposure.

In the present study, we analyzed the quantity of endotoxin and its relationship with the clinical findings, but we did not analyze the interventions needed to reduce exposure. However, our group has studied and published data obtained by applying questionnaires regarding the use of measures for the reduction of this exposure. Questions about the availability of breathing masks, glasses or protective visors, gloves and appropriate shoes for the workplace and about the use of this individual protection equipment (IPE) during contact with the animals revealed that the most accessible IPE were gloves (99 %) and the least accessible were appropriate shoes (36 %). Nineteen percent of animal handlers used breathing masks at all times while handling animals or when working in animal rooms, but only 7 % wore protective glasses and 24 % wore specific shoes [16].

The present results are very important for the understanding of the presence of endotoxins in the workplace and their interaction with workers’ symptoms, since there still are doubts about the role of endotoxins as a risk factor for respiratory diseases or as a protective factor against the latter.

## Conclusion

We conclude that exposure to endotoxin has an effect on the respiratory system of workers even though it is not directly associated with asthma. High endotoxin concentrations were associated with the presence of wheezing, especially in the group exposed to laboratory animals. This shows that animal house workers and workers who are in direct contact with laboratory animals are more susceptible to wheezing, with the need for preventive measures.

## Consent for publication

Not applicable.

## Availability of data and materials

Database is available on zenodo.org (https://zenodo.org/record/49686#.VxS4xPkrLIV).

## Abbreviations

95 % CI: 95 % confidence interval
BHR: bronchial hyperresponsiveness
CD14: cell differentiation marker
EU: endotoxin unit
FEV1/FVC: forced expiratory volume in one second/forced vital capacity
IL: interleukin
IPE: individual protection equipment
LAL: limulus amebocyte lysate
LPS: lipopolysaccharide
PR: prevalence ratio
UNICAMP: State University of Campinas
USP: University of São Paulo
